# Prediction models for essential thrombocythemia from two longitudinal studies involving 2000 patients

**DOI:** 10.1038/s41408-024-00987-y

**Published:** 2024-01-23

**Authors:** Tiziano Barbui, Alessandra Carobbio

**Affiliations:** FROM Research Foundation, Papa Giovanni XXIII Hospital, Bergamo, Italy

**Keywords:** Myeloproliferative disease, Risk factors

Over the past two decades, significant progress has been made in several areas of Philadelphia chromosome-negative myeloproliferative neoplasms (Ph-neg MPNs), namely polycythemia vera (PV), essential thrombocythemia (ET), and myelofibrosis (MF). The driver mutations in the JAK2-V617, MPL, calreticulin opened new diagnostic and prognostic possibilities and provided new targets for therapy [1].

ET is currently diagnosed according to the World Health Organization (WHO) [2] and International Consensus Criteria (ICC) [3] criteria, involving a comprehensive evaluation of clinical, laboratory and molecular features, and is defined by clonal thrombocytosis with characteristic bone marrow megakaryocyte morphology, which allows a differentiation from PV and prefibrotic myelofibrosis (pre-PMF); the latter is a distinct entity with a clinical picture often characterized by isolated thrombocytosis mimicking ET. In a multicenter series of 1104 patients previously classified as having ET, the diagnosis was re-evaluated following strict application of the 2008 WHO classification, which includes well-defined histopathological criteria. The diagnosis of ET was confirmed in 891 patients (81%) and revised to pre-PMF in 180 (16%) [4]. A subset of ET patients has a triple-negative (TN) genotype due to the absence of detectable mutations in driver genes and is observed in ~10% of ET cases [1].

Current information on risk factors of the major critical events (thrombosis, evolution to MF, blast phase (BP), and survival) derives from registry and multicenter observational studies while single-center reports conducted at tertiary referral institutions are very limited [5]. Each study design has its strengths and limitations. Observational multicenter studies and registries can capture a large number of cases but may face challenges related to data quality and consistency. Ensuring the accuracy and uniformity of data across multiple centers becomes a critical consideration.

Studies conducted in tertiary centers, exemplified by those presented in this Blood Cancer Journal issue from Florence [6] and Mayo Clinic [7] hospitals, are more robust in nature as they can provide a solid description of natural history of this myeloproliferative neoplasm. These are conducted by specialized teams comprising clinician hematologists, pathologists, geneticists, and other experts with proficiency in MPNs and are equipped with up-to-date technologies including molecular analyses, which are essential in the case of ET where genetic mutations play a significant role in diagnosis and prognosis. Nevertheless, despite providing comprehensive insights into a well-defined cohort of patients, these centers may have a patient referral bias and limited generalizability to the broader population. This may suggest that description of disease presentation and results on prognostic factors may not be universally reproducible, and caution should be exercised when extrapolating the results to consecutive patient groups.

The Mayo and Florence reports each included 1000 ET patients; all 2000 cases met ICC 2022 and WHO diagnostic criteria and were fully annotated for driver mutations; diagnosis required hematopathology review to minimize unintended inclusion of patients with masked PV or pre-PMF. This revision is critical for patients diagnosed with ET prior to the WHO recognition of masked PV and pre-PMF, as the incidence of complications such as thrombosis, myelofibrosis, blast phase, and overall survival differs between these entities compared to “true ET”. All patients in the two studies were annotated for driver mutations, which were found in approximately 90% of cases, with similar proportions in the two series for JAK2 V617F, CALR including CALR type 1/1-like and CALR type 2/2-like, MPL and TN. Interestingly, female sex clustered preferentially with TN and JAK2 vs. CALR/MPL mutations (*p* < 0.01), and extreme thrombocytosis clustered with CALR (type 2 more than type 1), TN, and MPL, whereas leukocytosis clustered with JAK2 mutation (*p* < 0.001). It is noteworthy that the two patients’ series from Mayo and Florence showed remarkably similar presentations over the extensive recruitment period of more than 40 years.

In these retrospective cohorts, 20% of patients had a history of vascular complications at diagnosis and a similar percentage of driver mutations clustered in a similar manner. Importantly, these findings are consistent with data observed in other real-world routine clinical practice of recent reviews on ET [8, 9]. This convergence of information on disease presentation between Mayo and Florence highlights that the characteristics of these two retrospective cohorts are unlikely to have been influenced by potential reference bias. Thus, the consistency of these patterns across different settings adds value to the findings of these two studies, reinforcing the reliability of the observed trends and minimizing the impact of referral bias.

Therefore, the Mayo and Florence longitudinal studies offer the unique advantage of capturing the dynamic evolution of ET disease in real-world clinical practice over an extended period of median 8.5 years (range, 0.01–52.7) and 8 years (range, 0.03–42.9), respectively, providing robust estimates of disease-specific outcomes, i.e., arterial and venous thrombosis, progression to overt MF, BP, and survival. This makes the results on risk factors for each of these critical events highly reliable and generalizable. In this context, the confirmation of the prognostic role of increased neutrophil granulocytes and decreased lymphocytes as independent risk factors for survival in 1164 ET patients should be highlighted. This new knowledge opens new avenues for future clinical trials on the role of inflammation in MPN and the associated new targets for therapy [10, 11]. In addition, the large number of cases annotated for driver mutations allowed the identification of risk scores for progression to myelofibrosis and blast phase and confirmed the predictive power of the International Prognostic Score of Thrombosis (IPSET-thrombosis) score. We agree with the authors that these results, obtained in a large series of patients with ET, mutually validated, can constitute a reference standard against which other series of cases fully annotated for driver mutations and followed up for a long time can be compared.

Inspired by the extensive ET series of these two Blood Cancer Journal papers, we reviewed our data on 891 WHO-diagnosed ET patients enrolled from multi-center institutions, in whom we investigated the effect of post-diagnosis intermediate events (thrombosis, MF, and BP) on mortality using multistate models [12]. Using these models, which increase the precision of estimation by correcting for competing risk factors, we found that patients with incident thrombosis had a progressively increased risk of death compared with patients without this event. As expected, the highest risk of death was associated with the occurrence of MF and BP (Fig. 1). Notably, in the time-dependent multivariate analysis, arterial but not venous thrombosis occurrence during follow-up was independently associated with death, together with evolution into MF and BP (Table 1). Therefore, in future analysis of longitudinal studies, we suggest that the conventional baseline prognostic evaluation in MPN should be revised by considering the intermediate events that might integrate the risk of the final outcome of interest in the single patient.

**Fig. 1:**
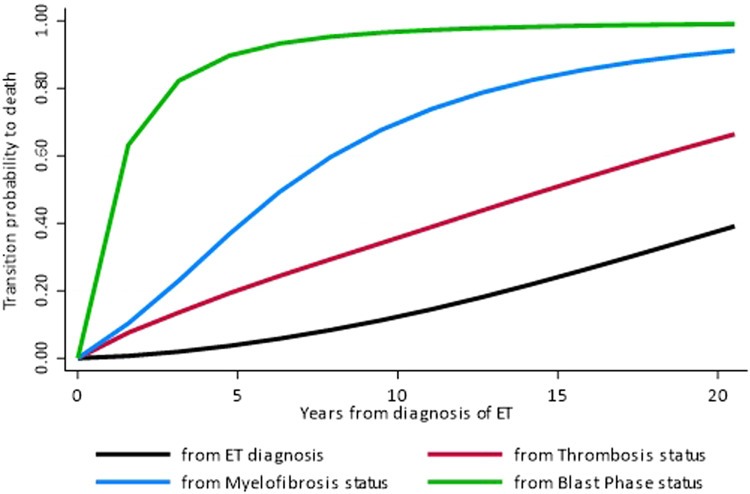
Transition probabilities to death in ET. Comparison of the direct (i.e., from ET diagnosis, black line) and indirect (i.e., via thrombosis [red line], evolution to MF [blue line] or BP [green line]) transition probabilities to death (absorbing state) over time in 891 ET patients. Transition probabilities are defined as the probability of going from a given state to death in a Markov process within a multistate model approach.

**Table 1:** Fixed and time-dependent risk factors for death prediction in 891 ET patients.

Covariates	HR	*p*	95% CI
*Fixed variables*			
Male sex	0.86	0.531	0.55–1.36
**Age** > **60 years**	**5.53**	**0.000**	**3.44**–**8.90**
JAK2V617F	1.30	0.560	0.54–3.14
**CV risk factors**	**3.04**	**0.000**	**1.88**–**4.92**
**Previous arterial thrombosis**	**2.09**	**0.002**	**1.31**–**3.36**
**Previous venous thrombosis**	1.32	0.362	0.72–2.43
*Time-dependent variables*			
**Arterial thrombosis**	**2.60**	**0.002**	**1.44**–**4.70**
Venous thrombosis	1.23	0.669	0.47–3.25
**Evolution to Myelofibrosis**	**4.35**	**0.002**	**1.69**–**11.20**
**Evolution to Blast phase**	**38.58**	**0.000**	**15.84**–**93.95**

Cox regression model adjusted for variables known at ET diagnosis (fixed effects) and time-dependent variables for events occurring during follow-up (variable effects) to identify risk factors for mortality. In bold statistically significant (i.e., *p* < 0.05) variables.

*HR* hazard ratio, *CI* confidence interval, *CV* cardiovascular.

In conclusion, these two comprehensive observational studies reflect real-world clinical practice in ET and provide both novel and confirmatory insights into its clinical features, mutational landscape, risk factors and potential therapeutic interventions. The identified associations and prognostic models provide a valuable foundation for future studies and guide further research into risk stratification and personalized management approaches for patients with essential thrombocythemia.

## Data Availability

Data are available on request due to privacy/ethical restrictions.

## References

[1] Tefferi A, Pardanani A (2019). Essential thrombocythemia. N Engl J Med.

[2] Khoury JD, Solary E, Abla O, Akkari Y, Alaggio R, Apperley JF (2022). The 5th edition of the World Health Organization classification of haematolymphoid tumours: myeloid and histiocytic/1 dendritic neoplasms. Leukemia.

[3] Arber DA, Orazi A, Hasserjian RP, Borowitz MJ, Calvo KR, Kvasnicka HM (2022). Internationa. Blood.

[4] Barbui T, Thiele J, Passamonti F, Rumi E, Boveri E, Ruggeri M (2011). Survival and disease progression in essential thrombocythemia are significantly influenced by accurate morphologic diagnosis: an international study. J Clin Oncol.

[5] Goulart H, Mascarenhas J, Tremblay D (2022). Low‑risk polycythemia vera and essential thrombocythemia: management considerations and future directions. Ann Hematol.

[6] Loscocco GG, Gesullo F, Capecchi G, Atanasio A, Maccari C, Mannelli F, et al. One thousand patients with essential thrombocythemia: the Florence- CRIMM experience. Blood Cancer J. 2024 (in press). 10.1038/s41408-023-00968-7 PMC10796728 38238287

[7] Gangat N, Karrar O, Al-Kali A, Begna KH, Elliott MA, Wolanskyj-Spinner AP, et al. One thousand patients with essential thrombocythemia: the Mayo Clinic experience. Blood Cancer J. 2024 (in press). 10.1038/s41408-023-00972-x PMC10796913 38238303

[8] Carobbio A, Ferrari A, Masciulli A, Ghirardi A, Barosi G, Barbui T (2019). Leukocytosis and thrombosis in essential thrombocythemia and polycythemia vera: a systematic review and meta-analysis. Blood Adv.

[9] Alvarez-Larran A, Sant’Antonio E, Harrison C, Kiladjian JJ, Griesshammer M, Mesa R (2021). Unmet clinical needs in the management of CALR-mutated essential thrombocythaemia: a consensus-based proposal from the European LeukemiaNet. Lancet Haematol.

[10] Hasselbalch HC (2012). Perspectives on chronic inflammation in essential thrombocythemia, polycythemia vera, and myelofibrosis: is chronic inflammation a trigger and driver of clonal evolution and development of accelerated atherosclerosis and second cancer?. Blood.

[11] Barbui T, Carobbio A, Finazzi G, Vannucchi AM, Barosi G, Antonioli E (2011). Inflammation and thrombosis in essential thrombocythemia and polycythemia vera: different role of C-reactive protein and pentraxin 3. Haematologica.

[12] Carobbio A, Vannucchi AM, Rumi E, De Stefano V, Rambaldi A, Carli G (2023). Survival expectation after thrombosis and overt-myelofibrosis in essential thrombocythemia and prefibrotic myelofibrosis: a multistate model approach. Blood Cancer J.

